# Reply to: Memory box: possibilities to support grief in the intensive care unit during the COVID-19 pandemic

**DOI:** 10.5935/0103-507X.20210045

**Published:** 2021

**Authors:** Thábata da Silva Cardoso Luiz, Orli Carvalho da Silva Filho, Tânia Cristina Camacho Ventura, Virginia Dresch

**Affiliations:** 1 Hospital Universitário Antônio Pedro, Universidade Federal Fluminense - Niterói (RJ), Brazil.; 2 Instituto Nacional da Saúde da Mulher, da Criança e do Adolescente Fernandes Figueira, Fundação Oswaldo Cruz - Rio de Janeiro (RJ), Brazil.; 3 Institute of Psychology, Universidade Federal Fluminense - Niterói (RJ), Brazil.

**Dear Editor,**

Regarding the comments related to the memory box intervention we described in our article,^(1)^ we are confident that this dialogue can qualify and value the transversality of mental health during the coronavirus 2019 disease (COVID-19) pandemic, as recommended by the World Health Organization (WHO) in a technical note issued shortly after this current pandemic condition was declared.^(2)^ In addition, we thank you for the opportunity to clarify these doubts related to this intervention, succinctly described in adherence to the format deliberated by the editorial board of this journal.

In response to both questions, we point out that the memory box was designed to be a device adapting the previously existing protocols for delivering dead patients’ hospital safeguarded belongings to families or guardians. In our settings, this delivery is an ethical and usual practice. Therefore there is no active search of objects, aiming to build a narrative, that would jeopardize the deceased one’s privacy. Therefore, in response to the second question, the memory box has not created a system for returning belongings, but qualified it instead, by providing a symbolic, respectful, and compassionate character.

The first question is very important, not only during the COVID-19 pandemic, as several nosocomial infection control protocols are in place to comply with good intensive care unit practices. Infection is possible through contact with surfaces contaminated with the severe acute respiratory syndrome coronavirus 2 (SARS-CoV-2). However, the current epidemiologic data and recommendations from health agencies, such as the Centers for Disease Control and Prevention (CDC), point out that transmission through surfaces is not the main form of dissemination, being this risk considered low.^(3)^ The main way in which the SARS-CoV-2 infection occurs is respiratory. The contamination risk can be reduced with the consistent use of masks, proper hand hygiene, and healthy facilities. Surface decontamination is recommended in closed community settings where a suspected or confirmed COVID-19 case was detected in the last 24 hours.^(3)^

As described in our report,^(1)^ the belongings are decontaminated and returned to the families in a timeframe greater than 72 hours, and the team meeting with family members adheres to safety protocols.

We emphasize that, at this time, clear and respectful communication of the intensive care unit teams with families and mourners, always adhering to safety rules, is configured as a fundamental health care strategy and, even during the pandemic, should be stimulated.^(4)^

We thank for the letter author’s interest and hope that there is interest and encouragement for different devices, such as the memory box, to be developed and refined, allowing for urgent incorporation of the mental health subject in the intensive care scenario.
